# Wandering behaviour prevents inter and intra oceanic speciation in a coastal pelagic fish

**DOI:** 10.1038/s41598-017-02945-0

**Published:** 2017-06-06

**Authors:** Gonçalo Silva, Regina L. Cunha, Ana Ramos, Rita Castilho

**Affiliations:** 1 Centre of Marine Sciences, CCMAR, University of Algarve, Gambelas, 8005-139 Faro Portugal; 2 MARE – Marine and Environmental Sciences Centre; ISPA - Instituto Universitário, Rua Jardim do Tabaco 34, 1149-041 Lisboa, Portugal

## Abstract

Small pelagic fishes have the ability to disperse over long distances and may present complex evolutionary histories. Here, Old World Anchovies (OWA) were used as a model system to understand genetic patterns and connectivity of fish between the Atlantic and Pacific basins. We surveyed 16 locations worldwide using mtDNA and 8 microsatellite loci for genetic parameters, and mtDNA (cyt *b*; 16S) and nuclear (RAG1; RAG2) regions for dating major lineage-splitting events within Engraulidae family. The OWA genetic divergences (0–0.4%) are compatible with intra-specific divergence, showing evidence of both ancient and contemporary admixture between the Pacific and Atlantic populations, enhanced by high asymmetrical migration from the Pacific to the Atlantic. The estimated divergence between Atlantic and Pacific anchovies (0.67 [0.53–0.80] Ma) matches a severe drop of sea temperature during the Günz glacial stage of the Pleistocene. Our results support an alternative evolutionary scenario for the OWA, suggesting a coastal migration along south Asia, Middle East and eastern Africa continental platforms, followed by the colonization of the Atlantic via the Cape of the Good Hope.

## Introduction

Periodic climatic events affect the evolution of the species, shaping their biogeographic and macroecological patterns^1^. Speciation in the marine realm is mostly related to geological and climatic events that have occurred throughout different periods^2, 3^. Pleistocene climatic events promoted fluctuations in sea surface temperatures, sea level, ice sheet coverage and changes on the global oceanographic circulation patterns, having impact on living species distribution, diversity, population structure and speciation (e.g. ref. 4). Contemporary changes on oceanographic features such as in frontal systems, upwelling events and environmental transitions may also have implications on species distribution patterns and life history traits (ref. 4 and references therein). Although cyclical periodicity of range shifts may enhance secondary contacts and prevent speciation^1^, the isolation experienced by some peripheral populations may also promote differentiation. Species that diverged during the Pleistocene often exhibit shallow divergence due to their recent isolation or incipient speciation^5^. The apparent absence of physical barriers in the marine environment creates additional difficulties to explain vicariant allopatric or peripatric speciation reported during this period^6^. Nonetheless, organisms often show limited distributional ranges imposed by intrinsic physiological constraints, dispersal ability or the incapacity to adapt to new environments. Throughout the Pleistocene climatic oscillations, organisms were able to cross temporarily interrupted barriers during specific periods of time^7^. Transitions over soft barriers include long-distance migrations through warm Equatorial waters (e.g. ref. 8) or interoceanic migrations (e.g. ref. 9).

**Figure 1:**
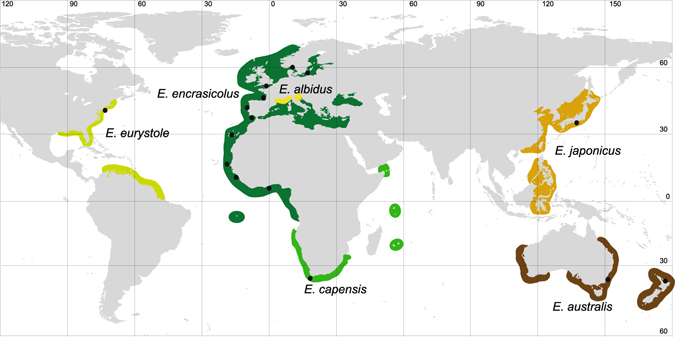
Present-day distribution of nominal Old World Anchovies species^10^; black dots represent sample locations; raw map was downloaded from https://freevectormaps.com/ and edited in Adobe Illustrator CS5.1 (Adobe Systems Inc., CA, USA).

Here, we used the Old World Anchovies (OWA) species complex^10^ as a case study of coastal pelagic fishes with high dispersal ability to analyse evolutionary relationships and the level of connectivity between the Atlantic and Pacific basins. The OWA are coastal fish species distributed along offshore areas above the continental platforms of the Atlantic and Pacific oceans, restricted sea basins in the Mediterranean Sea, the Baltic and the Black seas as well as inshore environments such as estuaries, inlets and bays^10^. This species complex comprises five nominal species: the Japanese anchovy *Engraulis japonicus* and the Australian anchovy *E. australis* in the western Pacific; the Cape anchovy *E. capensis* in the southeastern Atlantic Ocean; the silver anchovy *E. eurystole* in the western Atlantic Ocean; the European anchovy *E. encrasicolus* in the eastern Atlantic, Baltic Sea, Mediterranean Sea and the Black Sea. More recently, the white anchovy *E. albidus* was added to the OWA group, based on ecological, morphological and genetic differences between inshore and pelagic populations in the Mediterranean Sea^11^ (Fig. 1), but its specific status remains uncertain^12^. The original description of OWA nominal species was mainly based on traditional taxonomy and geography, with taxa longitudinally and latitudinally disjunct and hence classified as separated species^10^. Despite a large body of literature focusing on this group, the phylogenetic relationship between the OWA species remain poorly understood. This group is thought to have diverged recently from the remaining Engraulidae at about one million years ago^13^.

The OWA exhibit shallow genetic divergences between putative species and several conflicting aspects in the taxonomy and molecular classification of the group exist. Based on morphological characters, Whitehead and his colleagues^10^ proposed that the whole group should be considered a single species and molecular studies revealed that some of the described species have no genetic support. No significant genetic differences were found between *E. encrasicolus* and *E. eurystole* ^14^ and the existence of shared mtDNA haplotypes between *E. encrasicolus* and *E. capensis* ^15^ or between *E. australis* and *E. japonicus* ^16^, seriously compromise the current taxonomy of this group. The lack of genetic divergence among disjunct distributions contrasts with partial reproductive isolation and parallel genetic differentiation among sympatric ecological morphotypes^12, 17^ or with the complex population structure observed at regional spatial scales in the European anchovy^14, 18–22^. Also, *E. encrasicolus* mtDNA is divided in two lineages (clade A and B), but the paraphyly of these mtDNA lineages generated further debate^13–15^.

Pleistocene climatic swings influenced anchovies range shifts, lead to trans-equatorial dispersals during episodes of global cooling or through deep cold water^15, 16^ and promoted inter-oceanic migrations^16^. Anchovies are thought to have colonised the Atlantic Ocean through the southern Indian Ocean^16^. Anchovies in the Atlantic Ocean likely experienced several extinction-colonization cycles at the extremes of the distribution driven by Pleistocene climate shifts^14, 16^. Moreover, the western Atlantic was colonized after the last glacial maximum (LGM) from anchovies dispersing from western African populations of the European anchovy^14^. Recently, a mitochondrial-based analysis of *E. encrasicolus* (mitochondrial clade B) indicated that some of these individuals were found under selection, possibly related to adaptation to cold temperatures^23^. In the Pacific Ocean, the Japanese and Australian anchovies diverged between 105 ka (thousand years ago) and 420 ka^24^. The genetic signature of Pacific anchovies revealed persistence on separated hemispheres over several glacial cycles, although more recent dispersals were identified^16^. Thus far, studies involving OWA phylogenetic assessments were based on allozymes and on a small fragment of the mitochondrial cytochrome *b* gene (cyt *b*; 521 bp)^15, 16^.

In this study, we used a large portion (1044 bp) of the cyt *b* and 8 nuclear microsatellites (as described in Silva *et al*.^14^) to further analyse evolutionary relationships within OWA and provide a novel perspective on the level of connectivity between the Atlantic and Pacific anchovies. We dated main lineage splitting events within Engraulidae and propose a new biogeographic scenario for the OWA.

## Results

### Population structure, differentiation and connectivity

Multilocus genotypes from the 16 locations from the Atlantic Ocean and Pacific Ocean were obtained for 462 anchovies (Supplementary Fig. S1). The number of alleles per locus varied from 19 (locus Ee2–91b) to 85 (locus Ee10) over all locations (Supplementary Table S1). Mean allelic richness, standardized for comparison across a minimum common sample size of nine individuals, ranged from 6.9 (Senegal) to 9.3 (USA) in the Atlantic Ocean and from 7.4 to 9.4 in the Pacific Ocean (Table 1). POWSIM analysis indicated at least a 95% probability (based on the proportion of significant chi-squared tests) of detecting an *F*_ST_ value of as low as 0.001 using the microsatellite data set (Supplementary Fig. S2). Expected heterozygosity (*H*_E_) varied between 0.797 (Senegal) and 0.894 (USA) in the Atlantic Ocean and between 0.849 and 0.899 in the Pacific Ocean, while the observed heterozygosity (*H*_O_) varied between 0.653 (Guinea-Bissau) and 0.825 (Portugal north) in the Atlantic Ocean and between 0.699 and 0.742 in the Pacific Ocean (Table 1).

**Table 1:** Sample locations, sample abbreviations, collection dates, sample sizes and summary statistics for a 1044 bp sequence fragment of the mtDNA cytochrome *b* and eight nuclear microsatellites of Old World Anchovies (OWA).

Location	Code	Nominal species	Long	Lat	Year	Mitochondrial Cytochrome *b*				Microsatellites						
						*N*	*n*_h_	*h*	*π*	*N*	*Aavg*	*A*_r_ = 9	*A*_r_ = 18	*Effnum*	*H*_O_	*H*_*E*_
Norway	NO	*E. encrasicolus*	10.6	59.0	2007	24	17	0.953	0.009	40	13.13	7.38	10.25	6.973	0.714	0.850
Poland	PL	*E. encrasicolus*	16.5	54.6	2008	9	7	0.917	0.014	9	8.38	8.38	—	6.081	0.736	0.864
English Channel	EC	*E. encrasicolus*	0.1	50.8	2007	27	18	0.963	0.010	45	13.00	7.25	9.75	6.596	0.729	0.837
Bay of Biscay	BB	*E. encrasicolus*	−2.9	43.5	2007	23	19	0.980	0.015	45	16.00	7.88	10.38	6.926	0.762	0.837
Portugal - North	PN	*E. encrasicolus*	−8.8	40.7	1998	25	24	0.997	0.013	45	17.25	8.25	11.50	8.383	0.825	0.882
Portugal - South	PS	*E. encrasicolus*	−8.4	37.1	2007	29	27	0.995	0.011	43	17.88	7.63	12.63	8.588	0.777	0.880
Canary Islands	CA	*E. encrasicolus*	−15.0	28.3	1999	24	23	0.996	0.007	42	17.75	8.38	12.13	9.929	0.792	0.888
Senegal	SN	*E. encrasicolus*	−17.6	14.8	1999	25	25	1.000	0.006	37	12.50	6.88	9.38	4.973	0.693	0.797
Guinea-Bissau	GU	*E. encrasicolus*	−14.2	9.7	2006	20	20	1.000	0.006	19	13.13	8.38	12.88	6.751	0.653	0.868
Ghana	GH	*E. encrasicolus*	0.0	5.6	2008	25	25	1.000	0.006	27	15.50	8.88	12.88	8.664	0.766	0.883
Namibia	NM	*E. capensis*	11.7	−17.2	2007	24	17	0.920	0.010	32	14.38	8.88	11.63	8.375	0.769	0.875
South Africa	SA	*E. capensis*	21.0	−34.7	2007	13	10	0.923	0.019	21	12.63	9.13	11.50	8.148	0.768	0.887
USA	US	*E. eurystole*	−66.1	41.5	2006	12	9	0.909	0.004	18	13.50	9.25	13.50	7.978	0.793	0.894
Japan	JP	*E. japonicus*	139.9	35.6	2006	24	24	1.000	0.011	30	17.13	7.75	13.00	9.014	0.699	0.869
Australia	AU	*E. australis*	151.0	−35.0	2008	34	33	0.998	0.007	44	21.38	9.38	13.63	10.530	0.742	0.899
New Zealand	NZ	*E. australis*	175.0	−36.7	2005	35	15	0.709	0.001	34	14.38	7.38	11.38	6.771	0.717	0.849
**Total**						373	269	0.993	0.023	531	47.63	32.00	39.63	6.997	0.746	0.866

Long, longitude; Lat, latitude; *N*, number of individuals; *n*_*h*_, number of haplotypes; *h*, haplotype diverstity; *π*, nucleotide diversity; *Aavg*, average number of alleles, *Ar*, allelic richness; *Effnum*, Effective number of alleles; *H*_*0*_, observed mean heterozigozity; *H*_***E***_, expected mean heterozygozity.

**Figure 2:**
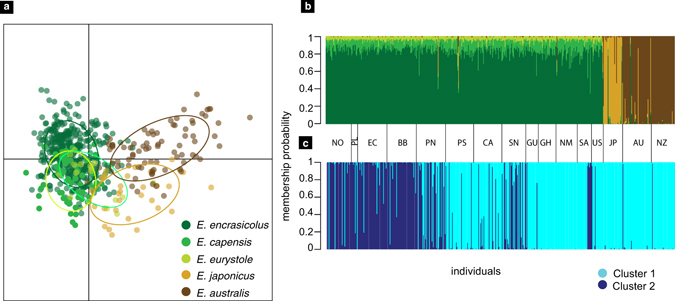
(**a**) Discriminant analysis of principal components (DAPC) of multi-locus Old World Anchovies genotypes; individual genotypes appear as circles; ellipses represent the centre of dispersion of each putative species (see Fig. 1 and Table 1). Horizontal and vertical axes are the first two principal components, respectively; (**b**) scatter plot of *a priori*-defined nominal species; (**c**) individuals assigned to their genetic cluster without forcing them into pre-determined groups.

The DAPC results show that *E. encrasicolus*, *E. eurystole* and *E. capensis* are closer to each other than to the *E. australis* and *E. japonicus* clusters (Fig. 2a). The probability of assignment of individuals to their nominal species (Fig. 2b) shows that *E. eurystole* and *E. capensis* individuals were mostly assigned to the *E. encrasicolus* clusters with probabilities close to 0.8, and *E. australis* and *E. japonicus* were mostly assigned to their own clusters also with high probabilities. The *a posteriori* DAPC analysis (Fig. 2c) shows two clusters, one comprising 167 *E. encrasicolus* individuals, and all *E. eurystole, E. capensis, E. australis* and *E. japonicus*, and another with 157 *E. encrasicolus*.

Estimates with microsatellite data of the mutation scaled population size parameter were higher in the Pacific Ocean than in the Atlantic Ocean (Θ_Atlantic_ = 11.6 and Θ_Pacific_ = 98.4). Our comparison of candidate models of gene flow between populations, clearly rejects panmixia, and showed that migration from the Pacific to the Atlantic (model 2) fitted our microsatellite data best (Table 2), but with extremely low gene flow (*M* = 1.5).

**Table 2:** Bayes factors model comparison of migration models for Old World Anchovies between the Atlantic (ATL) and the Pacific (PAC) oceans.

Marker	Models		Model parameters	Bézier	dBézier	Probability
mtDNA	Model 1	ATL <−> PAC	****	−5339.10	0.00	1
	Model 2	ATL <− PAC	*0**	−5396.31	−57.20	1.4342E-25
	Model 3	ATL −> PAC	**0*	−5375.88	−36.77	1.071E-16
	Model 4	(ATL + PAC)	*0*0	−5524.26	−185.15	3.87457E-81
Microsatellites	Model 1	ATL <−> PAC	****	−329230.64	−232312.46	0
	Model 2	ATL <− PAC	*0**	−96918.17	0.00	1
	Model 3	ATL −> PAC	**0*	−205619.62	−108701.45	0
	Model 4	(ATL + PAC)	*0*0	−384554.60	−287636.43	0

Model parameters code as follows: asterisk (*) indicates that a particular migration rate was estimated by the model and 0 indicates that no migration was allowed. The first sign indicates theta for Atlantic, the second sign migration to Atlantic, the third sign theta for Pacific and the forth sign migration to Pacific.

A total of 373 individuals from five OWA putative species and 16 locations were analysed for mitochondrial cyt *b* gene, yielding 269 haplotypes. Haplotype diversity (*h*) was generally high, ranging from 0.917 to 1.000 in the eastern Atlantic Ocean (from Norway to South Africa), 0.909 in the western Atlantic, and from 0.709 to 1.000 in the Pacific Ocean (Table 1). Nucleotide diversity (*π*) was low, ranging from 0.4% (USA) to 1.9% (South Africa) in the Atlantic Ocean and from 0.1% to 1.1% in the Pacific Ocean (Table 1). Evolutionary net divergence between OWA putative species varied between 0–0.4% (Table 3).

**Table 3:** Estimates of net evolutionary divergence between putative species of Old World Anchovies (below diagonal) and standard error values (above the diagonal).

	*E. encrasicolus*	*E. capensis*	*E. eurystole*	*E. japonicus*	*E. australis*
*E. encrasicolus*		0.000	0.001	0.000	0.003
*E. capensis*	0.000		0.001	0.000	0.003
*E. eurystole*	0.001	0.001		0.001	0.003
*E. japonicus*	0.000	0.000	0.001		0.002
*E. australis*	0.003	0.003	0.004	0.002	

**Figure 3:**
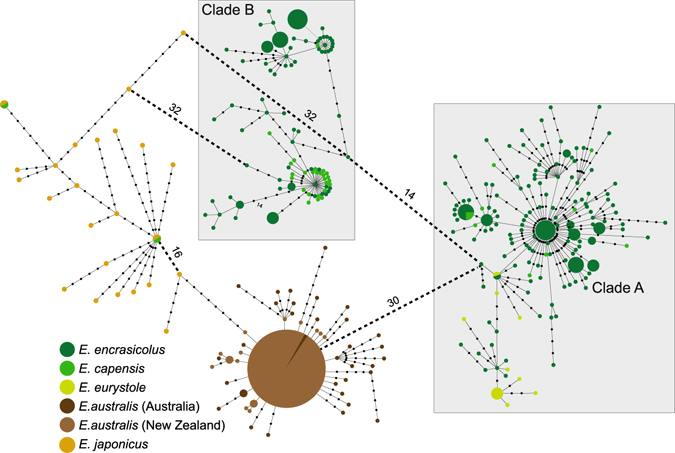
Minimum-spanning tree for the Old World Anchovies, based on the mitochondrial cytochrome *b* (1044 bp, 373 individuals). The colour and the size of the circles represent the geographic source (according to Fig. 1, with the exception of New Zealand haplotypes which are represented here in light brown) and frequency of each haplotype, respectively. The smallest colored circles represent a singleton haplotype. Black circles represent either extant unsampled sequences or extinct ancestral sequences. The length of the lines connecting haplotypes is proportional to the number of mutational differences separating the haplotypes, except when otherwise indicated.

The haplotype network revealed four main clades, two in Atlantic Ocean and two in the Pacific Ocean (Fig. 3). Clades from the two oceanic basins were separated by a minimum of 30–32 mutations, with two individuals from South Africa clustering within the northern Pacific clade. Within the Atlantic Ocean, putative species *E. encrasicolus*, *E. capensis* and *E. eurystole* were not reciprocally monophyletic, but alternatively fit on European anchovy clades A and B. The southern Pacific clade includes haplotypes mostly from *E. australis*, but also two haplotypes from *E. japonicus* that are separated nine mutations from the most frequent haplotype of this clade. These likely represent intermediate haplotypes between the two clades. The four clades exhibited different haplotype patterns: *E. encrasicolus* clade A and *E. australis* were characterized by multiple star-like radiations with relatively shallow genetic divergences; *E. encrasicolus* clade B and *E. japonicus* lacked distinct star patterns and exhibited possibly unsampled or extinct haplotypes (Fig. 3). Pairwise-taxa and pairwise-location *F*_ST_, *G*_ST_ and *D*_EST_ indicated that genetic differentiation among putative species was low, with the Atlantic putative species (*E. encrasicolus*, *E. capensis* and *E. eurystole*) and the japanese anchovies (*E. japonicus*) showing the lowest genetic differentiation between sea basins (Supplementary Fig. S3).

Estimates with mtDNA data of the mutation scaled population size parameter were the same between ocean basins (Θ = 0.1). Our comparison of candidate models of gene flow between populations, clearly rejects panmixia and showed that the full gene flow model (model 1) fitted our mtDNA data best (Table 2), with highly asymmetrical immigration between ocean basins, with the Pacific population providing five times as many immigrants into the Atlantic Ocean than vice-versa (*M* = 14.5 *vs*. 2.9, respectively).

### Phylogenetic analyses and Divergence Time Estimates

**Figure 4:**
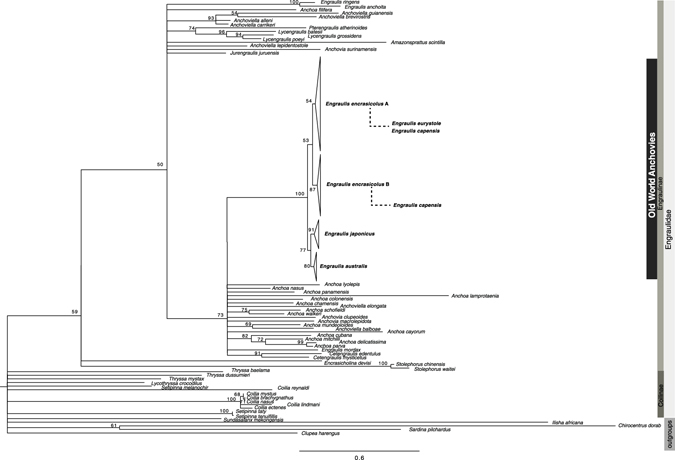
Phylogenetic relationships of Old World Anchovies *Engraulis spp*. maximum likelihood analyses inferred from a fragment of 1044 bp of cyt *b*. Maximum likelihood bootstrap values for major supported clades larger 50% are shown above branches.

The resulting topologies inferred from the BI and ML analyses based on the two data sets (with and without the mutation in the codon 368 of the analysed portion of cyt *b*) were identical (Fig. 4 and Supplementary Figs S4 and S5). Bayesian (−ln *L* = 21,088.60) and ML (−ln *L* = 20,986.77) analyses arrived at similar topologies (Fig. 4 and Supplementary Fig. S4, respectively) and therefore BI is shown in Supplementary Information (Supplementary Fig. S4). Potential Scale Reduction Factors in the BI analysis were about 1.00 and the average ESS value for all parameters (1385.01) was significantly larger than 200, which indicates convergence of the runs. In ML analysis (Fig. 4), four main monophyletic groups were retrieved within the OWA *Engraulis* spp. complex corresponding to *E. japonicus*, *E. australis*, a third clade including specimens assigned to *E. encrasicolus* clade A and a fourth clade including specimens assigned to *E. encrasicolus* clade B. *Engraulis eurystole* specimens grouped within *E. encrasicolus* clade A, and *E. capensis* specimens grouped both within clade A and B of *E. encrasicolus*. *Engraulis encrasicolus* clades A and B were well supported in both analyses (clade A: Bayesian posterior probabilities, BPP = 87, and ML bootstrap proportions, BP = 59; clade B: BPP = 100, BP = 89). Phylogenetic relationships within Engraulidae, either with ML or BI approaches, were generally unresolved and it was not possible to confidently establish the sister group of the *Engraulis* spp. complex (Fig. 4 and Supplementary Fig. S4).

**Figure 5:**
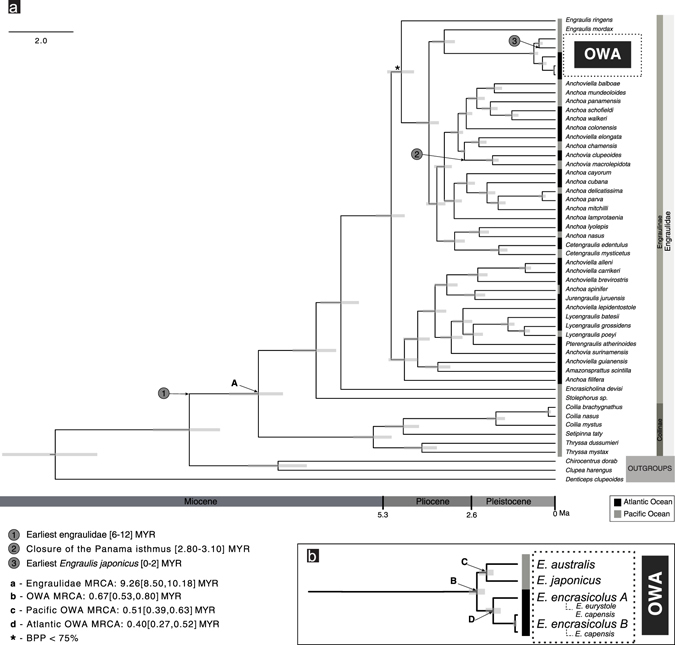
(**a**) Bayesian dating analysis of the Engraulidae family based on the concatenation of mitochondrial (cytochrome *b*: 1131 bp and 16S rRNA: 800 bp) and nuclear (RAG1: 1480 bp and RAG2: 1221 bp) gene fragments; (**b**) inset of the Old World Anchovies (OWA); numbers (1) and (3) are fossil calibrations, while number (2) represents a geologic event calibration; (A) (B) (C) (D) are relevant nodes ages; all branches show BPP > 75% except the node signed with * (53%).

Beast dating analysis based on the concatenated data set of mtDNA (cyt *b* and 16S) and nuclear introns (RAG1 and RAG2) fragments of the family Engraulidae estimated the age of the most recent common ancestor (MRCA) of the OWA at 0.67 Ma ago [0.53–0.80] (Fig. 5). The age of the MRCA of the Atlantic anchovies was estimated at 0.40 [0.27–0.52] Ma, while the split of the Pacific anchovies was estimated at about 0.51 [0.39–0.63] Ma.

## Discussion

Genetic divergences detected between Atlantic and Pacific anchovies are more compatible with intraspecific divergence (net divergence = 0.2%; Table 3) and indicate ongoing gene flow between oceanic basins. These results suggest that reproductive isolation between OWA putative species is not complete, and revealed the existence of regional variants or incipient species. The reconstructed genetic patterns within the OWA species complex found in the present study are in agreement with the findings of Whitehead and his colleagues^10^ based on morphological characters that consider the OWA as a single species. Regardless of the increased sequence length and the addition of more taxa, the precise origin of the OWA remains uncertain. The colonisation of the Atlantic Ocean likely occurred via South Africa, with anchovies dispersing across the northern Indian Ocean along the continental platforms of South Asia, Middle East and eastern Africa. The Atlantic and Pacific OWA revealed independent demographic histories but with contemporary gene flow. Before addressing the main interpretations and conclusions of these results, two main caveats must be addressed. First, data from the Pacific Ocean rely on only three sampling sites. Second, despite the rare records in the eastern African coast, no intermediate locations were sampled between the Atlantic and the Pacific oceans, thus we may have a restricted representation of the genetic diversity from the Indo-Pacific region. Nevertheless, the results presented in this study provide compelling evidence to set a biogeographical scenario for the OWA.

Previous phylogenetic analyses based on mitochondrial data (cyt *b*: 521 bp) recovered two lineages (clades A and B) within *E. encrasicolus* that did not group together^13, 15, 16^. The assumption of paraphyly for this species was uniquely based on a neighbour joining analysis that grouped the clade B with *E*. *japonicus* with relatively low statistical support (BP 53%). A more recent study showed that some individuals belonging to the *E. encrasicolus* mitochondrial clade B were under selection^23^ and therefore it is pivotal to understand if selection plays an important role in shaping the evolutionary and demographic history of this complex. All phylogenetic analyses (ML and BI) presented here that included more sequences (55 Engraulidae species) and a larger fragment (1044 bp) than previous studies, returned very similar results, regardless the effect of selection over the analysed portion of the cyt *b*. Moreover, unlike previous results, our ML analyses retrieved *E. encrasicolus* as monophyletic (Fig. 4), while the BI results do not reject it (Supplementary Fig. S4).

Our Bayesian dating analysis estimated that the divergence between the Pacific and the Atlantic OWA occurred during the Pleistocene at 0.67 Ma (Fig. 5). This divergence is more recent than previously estimated^13, 16^. Grant and his colleagues^13^ used a fixed mutation rate of 1.9%/Ma based on the separation between *Cetengraulis edentulus* and *C. mysticetus* determined by the closure of the Panama seaway. We also used the closure of the Panama Isthmus as a calibration event but added two fossil calibrations that were modelled with a lognormal distribution, which may explain the differences between age estimates from both studies.

Our results indicated that the OWA coalesced during the late Pleistocene (Fig. 5). Despite the few observed exceptions, such as those observed e.g. in the genus *Gobiodon* ^25^ that are compatible with a Pleistocene divergence time frame [0.01–2.60 Ma], most contemporary reef fishes show an earlier origin back to the Pliocene [2.60–5.30 Ma] or Miocene [5.30–23 Ma] (reviewed in ref. 5). Within the Pacific Ocean, *E. japonicus* and *E. australis* diverged at 0.51 [0.39–0.63 Ma] (Fig. 5), earlier than previously estimated [0.11–0.42 Ma]^24^.

To the best of our knowledge, values of genetic divergence between OWA putative species based on cyt b (0.0–0.4%; Table 3) constitute the lowest percentage of sequence divergence reported for intrageneric fish species^26^. Microsatellite data also showed a high degree of admixture between putative species divided into two clusters (Fig. 2c) that largely coincide with the northern part of the Atlantic Ocean (*E. encrasicolus* and *E. eurystole*) and the southeastern Atlantic and Pacific Oceans (*E. encrasicolus*, *E. capensis*, *E. japonicus* and *E. australis*). The DAPC (Fig. 2) also shows the existence of contemporary gene flow between anchovies from the Atlantic and Pacific basins. Low intraspecific genetic differentiation between individuals from distant locations was also detected on other coastal fish species^27–29^. The climate oscillation on relatively short time scales from decades to hundreds of thousands of years promotes shifts in distribution ranges, abundance (ref. 4 and references therein) and cyclical population extinctions and recolonizations^30^ that prevent the formation of deep lineages in small pelagics such as sardines and anchovies^31^.

Our results do not unequivocally support the origin of the OWA in the Pacific Ocean as no clear sister group of the OWA emerges from the ML and BI phylogenetic analyses of the cyt *b* region (Fig. 4 and Supplementary Fig. S4). Also, we cannot conclude if the Atlantic OWA resulted from a single colonization event or if they are the product of more than one invasion on different timescales^16^ given the low statistical support of the node corresponding to the Atlantic MRCA in all phylogenetic trees retrieved with different methods (Fig. 4 and Supplementary Fig. S4). Regardless the number of colonisations, the Pacific anchovies may have reached the Atlantic Ocean by three possible colonization pathways: (1) via Cape Horn in South America, (2) across the Bering strait in the Arctic Ocean, and/or (3) coastal dispersal throughout the Indian Ocean via the Cape of Good Hope in South Africa.

Dispersal through the Cape Horn was unlikely because the OWA do not occur on the coastline of South America and there are no paleontological records in the area of species belonging to this group. Colonization of the Atlantic Ocean through the Bering Strait would only have been possible during an interglacial period when the ice sheets melted and temperatures raised up between 0 and 2 °C. However, the estimated origin of their MRCA occurred at 0.67 Ma (Fig. 5), which coincided with a glacial period. Moreover, OWA from the north Atlantic and north Pacific are not genetically close (Figs 2 and 3; Supplementary Fig. S3). The third hypothetical dispersal route from the Pacific to the Atlantic is via the Cape of the Good Hope in South Africa. There is evidence supporting this alternative route around the South Africa gateway: (1) morphological similarities between Pacific and Atlantic Ocean OWA and (2) the predominant direction of OWA migration is from the Pacific to the Atlantic Ocean (Table 2). This dispersal most likely occurred throughout an interglacial period. The estimated divergence that occurred at 0.67 Ma (OWA MRCA; Fig. 5) between anchovies from the Indian and the Atlantic waters was probably promoted by the Günz glacial period that prevented dispersal between the two ocean basins. Studies on the evolution of the OWA performed thus far already suggested this route as the most likely for the Atlantic colonisation^15, 16, 31^. Grant and his colleagues^15, 16^ postulated that anchovies used this migration path through open-ocean, via temperate Indian Ocean and South Africa. These authors considered that this route would be the only possible colonisation pathway for temperate-water species as occurs with other small pelagic fish (e.g. sardines)^31^, given the high sea surface temperatures (SST’s) in the coastlines of eastern Africa and southern Asia. Until now, few records of OWA were reported in warmer habitats^10^, but recent studies indicated that higher SST’s are not a limiting factor for the OWA, as they can be found in tropical waters both in the Atlantic and Pacific oceans^10, 14, 23^. The colonisation pathway proposed by Grant and Bowen^16^ would imply more than 8000 km of open-ocean migration between the western Australia and South Africa without any stepping-stones. Anchovies are coastal pelagic fishes that live in average up to three years and it would be extremely unlikely that anchovies survived to a trans-oceanic migration of this magnitude in a single generation with no stopovers^32^. Open-ocean areas usually exhibit low productivity and scarce food resources^33^, which would create additional difficulties to large-scale migrations. Moreover, anchovies from Australia and southeastern Atlantic are not closely related in any of the phylogenetic analyses performed thus far (Figs 2 and 3)^15, 16^.

Alternatively, we propose a dispersal route of the OWA from the Pacific to the Atlantic across the continental platforms of the northern Indian Ocean, eastern African coast and South Africa, impelled by the North Equatorial and Agulhas Currents (Supplementary Fig. S6). The existence of shared microsatellite alleles (Fig. 2) and haplotypes (Fig. 3) between Atlantic and Pacific OWA revealed by our data, points to the existence of recurrent migration events and contemporary gene flow between ocean basins. We propose that the Atlantic colonisers were likely seeded from an Indo-Pacific pool that could have included both north and south Pacific anchovy ancestors, departing from Philippines and Indonesia, using Somalia, Mauritius and Seychelles as stepping-stones^10^. Pleistocene colonisations from the Pacific to the Atlantic through the Cape of the Good Hope were inferred for other coastal fish, mostly for tropical species (e.g. ref. 32). Trans-oceanic dispersals of tropical fish between the Indian and the Atlantic oceans are generally conditioned by the cold Benguela upwelling in the western South Africa, but fluctuations on the intensity of this current along the Pleistocene allowed punctuated episodes of dispersal^34^. Apparently, trans-oceanic dispersal of OWA from the Indian to the Atlantic Oceans is not limited by the Agulhas and Angola warm currents, nor the Benguela cold current due to the apparent wide temperature range tolerance of anchovy species. Climatic fluctuations of mid/late Quaternary are likely to have contributed to anchovies demographic expansions/contractions as seen in other species (ref. 4 and references therein) leading to extinction-colonisation cycles at the extreme of the distribution areas and to population connectivity/ differentiation.

## Conclusions

Comprehensive information on coastal fish genetic diversity and dispersal provides a wide-scale perspective of marine species connectivity and evolution. The results of our survey of the OWA complex *Engraulis spp*. here highlight that (i) coastal fish may disperse along large distances and frequently cross biogeographic barriers while maintaining high levels of genetic diversity and low genetic differentiation among populations; (ii) the dispersal route from the Pacific to the Atlantic Ocean is shared by other coastal fish species (e.g. ref. 32), increasing the support for a common model of intra-specific long distance dispersal; (iii) the permeability of biogeographical barriers depends on the relaxation of environmental conditions of ocean fronts; (iv) differentiation patterns depend on an intricate relationship between common geographic distances among populations, main circulation patterns, punctuated effectiveness of the biogeographic soft barriers promoted by climate oscillations and on ecological and biological traits at intraspecific level.

## Material and Methods

### Ethics statement

No specific permits were required for the field studies described here, since fish were purchased at fish markets or were collected on scientific cruises with fishing procedures. We confirm that the study locations were not privately owned or protected, and the field sampling activities did not involve endangered or protected species beyond the focal species.

### Sample collection, DNA extraction and PCR amplification

Samples of OWA likely representing five putative species were collected from 16 locations from both Atlantic and Pacific oceans (Fig. 1 and Table 1). The identification of the sampled specimens was based on their geographical origin, given the lack of morphological differentiation and diagnosing characters between putative species^10^. Fish were purchased at small coastal fish markets, as artisanal fishermen do not venture far, or were collected on scientific cruises (see acknowledgements). A small portion of white muscle or fin was preserved in 96% ethanol and stored at −20 °C. Total DNA extraction, polymerase chain reaction (PCR), purification of the PCR product, sequencing for a fragment of the mitochondrial cyt *b* (1044 bp) and microsatellite genotyping of eight loci were performed as described in Silva *et al*.^14^. Sequences of the nuclear recombination-activating genes RAG1 (1480 bp) and RAG2 (1221 bp) and of the mitochondrial 16S rRNA (800 bp) from OWA putative species used in the Bayesian dating analysis were obtained as described in Bloom & Lovejoy^35^.

### Sequence alignment, population structure, differentiation and connectivity

To determine contemporary genetic structuring and individual assignments based on the autosomal microsatellite data set, we used discriminate analysis of principal components (DAPC) implemented in the adegenet package^36^ of R 2.15.3^37^ and following Silva *et al*.^14^. DAPC was chosen over Bayesian clustering methods because this method is model free does not assuming Hardy–Weinberg equilibrium or linkage disequilibrium being more appropriate for situations where such assumptions are not met, as is often the case with anchovies^38^.

The program POWSIM 4.0^39^ was used to evaluate statistical power for detecting pairwise genetic differentiation at *F*_ST_ levels ranging from 0.00 to 0.10. We simulated the divergence of three subpopulations, corresponding to (1) European anchovy (*E. encrasicolus*, *E. capensis* and *E. eurystole*), (2) *E. japonicus* and (3) *E. australis*, from a single ancestral population through genetic drift to a given overall *F*_ST_ value defined by controlling effective population size (*N*e) and number of generations (t). To best reflect the assumingly large *N*e of anchovy, we let *N*e = 10 000 and varied *t* from 0 to 2078 for simulating different levels of differentiation. After the simulation, each subpopulation was sampled at n = 381 and divergence from genetic homogeneity was tested with ***χ***-exact test. This procedure was repeated 100 times and the proportion of significant outcomes was used to estimate statistical power for detecting pairwise genetic differentiation.

Cyt *b* sequences were aligned using ClustalX 2.0.3 with default settings, implemented in Geneious 5.4^40^, checked and trimmed manually. Sequences were reduced to haplotypes using Collapse 1.2^41^. Number of individuals (*N*), number of haplotypes (*n*_*h*_) and haplotype (*h*) and nucleotide diversities (*π*) were calculated in Arlequin 3.5.1.2^42^ using the cyt *b* data set.

Summary statistics, number of individuals (*N*), average number of alleles (A_avg_), observed heterozygosity (*H*_O_) and expected heterozygosity (*H*_E_) were calculated for each location and for each locus with Genodive^43^. Net evolutionary divergence between putative species of OWA was calculated on MEGA 5^44^ using the Maximum Composite Likelihood model. The rate variation among sites was modelled with a gamma distribution (shape parameter = 1.48).

To examine the relationship between mitochondrial haplotypes, a minimum spanning network was constructed with Arlequin 3.5.1.2^42^ and visualized with Hapstar ^45^. Pairwise genetic differentiation was estimated with *G*_*st_est*_^46^ and Jost’s *D*_*est*_ value^47^, both within and between putative species, following Pennings *et al*.^48^ for mtDNA and using the R package Diversity ^49^ for microsatellites.

### Estimation of migration rates

We used the coalescent-based program Migrate-N^50, 51^ to compare different biogeographic hypotheses for the past and present migration of OWA between the Atlantic and the Pacific oceans. We conducted the analyses with two sets of data, mitochondrial DNA and microsatellites, structured into two groups according to geographical regions: southern Atlantic Ocean (pooled southern locations Senegal, Guinea-Bissau, Ghana, Namibia, Angola and South Africa) and Pacific Ocean (Japan, Australia and New Zealand). We tested four variations of the two-population (Atlantic-Pacific) migration model: bidirectional migration (full model, four parameters), strict Atlantic to Pacific migration (three parameters), strict Pacific to Atlantic migration (three parameters) and panmictic model that assumes the Atlantic and Pacific are part of a panmictic population (one parameter). Testing the directionality of gene flow is justified because the dominant ocean current between the ocean basins, the Agulhas flow, runs westerly from the Indian to the Atlantic Ocean and is thought to play a limiting role in marine dispersal in the opposite direction^52, 53^. Initial values were calculated using *F*_ST_. Mutation rates were set to be constant among loci. The Migrate-N run parameters were calibrated on the full model for convergence of the Markov chain Monte Carlo sampling method. The prior distributions were uniform for mutation-scaled population size parameters theta (θ), that are four times the product of the effective population size and the mutation rate, and mutation-scaled migration rates M, that is, immigration rate scaled by the mutation rate, over the range of θ = 0.05–0.5 and prior migration rate M = 0–100 for mtDNA, and θ = 10–100 and M = 0–50 for microsatellites. These settings resulted in converged posterior distributions with a clear maximum for each estimate. The Bayesian run for mtDNA consisted of one long chain with a total of 15 million states visited and 50,000 states recorded for the generation of posterior distribution histograms for each locus after discarding the first 10,000 genealogies as burn-in; for all loci, a total of 48 million states were visited and 160,000 samples were recorded. For all the analyses we used an adaptive heating scheme with four simultaneous chains using different acceptance ratios (temperature settings were 1.0; 1.5; 3.0; 1 × 10^6^); the analyses were run on a cluster computer using 4 compute nodes per run. The Bayesian run for microsatellites consisted of one long chain with a total of 6 million states were visited and 20,000 states were recorded for the generation of posterior distribution histograms for each locus after discarding the first 10,000 genealogies as burn-in; for all loci, a total of 48 million states were visited and 160,000 samples were recorded. For all the analyses we used an adaptive heating scheme with four simultaneous chains using different acceptance ratios (temperature settings were 1.0; 1.5; 3.0; 1,000,000.0); the analyses were run on a cluster computer using four compute nodes per run. Overall loci information was combined into a single estimate by Bézier approximation of the thermodynamic scores as described by Beerli & Palczewski^54^. We averaged the Bézier score over three different runs and used as input to evaluate multiple models using Bayes factors^55^.

### Phylogenetic and dating analyses

Phylogenetic relationships within Engraulidae were based on a fragment of the mitochondrial cyt *b* gene (121 taxa, corresponding to 55 Engraulidae species; 1044 bp). At least one representative species of Engraulidae per genus (except *Papuengraulis*) and nine randomly chosen specimens from each of the five currently recognized OWA species were included in the phylogenetic analyses, with the exception of *E. capensis* from which only four specimens were available (accession numbers in Supplementary Table S2). According to Lavoué *et al*.^56^, we selected the following outgroup species: *Chirocentrus dorab*, *Clupea harengus*, *Denticeps clupeoides*, *Ilisha africana*, *Sardina pilchardus*, *Sundasalanx mekongensis*. The Akaike Information Criterion (AIC)^57^ implemented in Modeltest 3.7^58^, selected the GTR+I+Γ as the evolutionary model that best-fitted the data set. The inferred parameters were used in maximum likelihood (ML) and Bayesian Inference (BI) analyses. BI analyses were conducted with MrBayes 3.2.1^59^. Metropolis-coupled Markov chain Monte Carlo (MCMC) analyses were ran for 20,000,000 generations with sample frequency of 2000. Final trees were calculated after a burnin of 1,000 generations. PhyML 3.0^60^ was used to estimate the ML tree and to test by non-parametric bootstrapping the robustness of the inferred trees using 1,000 pseudoreplicates.

Previous work^15, 16^ recovered *E. encrasicolus* as paraphyletic (specimens assigned to the species grouped into two clades that did not group together). To test if natural selection could interfere with phylogenetic inference, we performed all phylogenetic analyses using the above data set that only included individuals that do not show any evidence of being under selection and repeated the procedures using another data set (116 taxa; 1044 bp) that included individuals presenting a mutation in codon 368 of the cyt *b* as identified in Silva *et al*.^23^.

To estimate the OWA origin and date lineage-splitting events within Engraulidae we used a Bayesian relaxed molecular-clock approach as implemented in Beast 2.1.3^61^ based on a concatenated dataset of four partial fragments of mtDNA (cyt *b*: 1131 bp; 16S: 800 bp) and nuclear (RAG1: 1480 bp; RAG2: 1221 bp) genes. We included sequence data of 49 Engraulidae lineages/taxa^35^, likely representing 14 genera (out of 16: the monospecific *Lycothrissa* and *Papuengraulis* genera are not represented), from which 5 are OWA (accession numbers in Supplementary Table S3). According to our ML and BI analyses, *E. capensis* and *E. eurystole* are conspecific with *E. encrasicolus*, and two clades (hereafter clade A and clade B) were recovered within the latter. Hence, to perform the dating analysis we only selected a single representative of *E. encrasicolus* from clade A and two from clade B, both without the mutation at codon 368. We used the BirthDeath model for the tree prior that assumes that at any point in time, every lineage undergoes speciation at rate λ or goes extinct at rate μ^62^, and three calibration points. One refers to the earliest record of Engraulidae [6–12] million years (Ma) from the Miocene - Lower Pliocene of Cyprus^63^. The second calibration corresponds to age estimated for the divergence between *Anchovia clupeoides* and *A. macrolepidota* [2.8–3.1] Ma due to the closure of the Panama seaway^13, 64^. The third calibration corresponds to *E. japonicus* [2–0] Ma from Kokubu group, Japan^65^. Calibrations using the two fossils were modelled with a lognormal distribution, where 95% of the prior weight fell within the geological interval in which each fossil was discovered. For the Engraulidae [12–6] Ma, the parameters of the lognormal calibration prior were: 95% interval: mean in real space: 1.4, offset: 6.0 and log stdev: 1.0. For *E. japonicus* [2–0] Ma, the parameters of the lognormal calibration prior were: 95% interval: mean in real space: 0.465, offset: 0 and log stdev: 1.0. For the divergence between *Anchovia clupeoides* and *A. macrolepidota* we used a calibration according to Lessios *et al*.^64^ where the closure of the isthmus of Panama occurred between 3.1–2.8 Ma. Lognormal calibration was set to: 95% interval: mean in real space: 0.071, offset: 2.8 and log stdev: 1.0. MCMC analyses were run for 20,000,000 generations with a sample frequency of 20,000, following a discarded burn-in of 2,000,000 steps. The convergence to the stationary distributions was confirmed by inspection of the MCMC samples using Tracer 1.6^66^.

ML, BI and dating analyses were performed on the R2C2 research group cluster facility provided by the IT Department of the University of Algarve.

### Data Accessibility

Sequences of the mitochondrial cyt *b* (1044 bp) were deposited in GenBank (accession numbers: JQ716609–JQ716731, JQ716748–JQ716756, JX683020–JX683113, KF601435–KF601478 and KJ007642–KJ007734). Sequences of the nuclear recombination-activating genes RAG1 and RAG2 and of the mitochondrial 16S rRNA used in the Bayesian dating analysis were deposited in GenBank (accession numbers: KX824115–KX824124). The accession numbers of the sequences retrieved from GenBank corresponding to the remaining Engraulidae specimens used in all phylogenetic analyses are indicated in Tables S2 and S3 (SI). Genotypes of individuals obtained from microsatellites are deposited in Dryad repository (http://dx.doi.org/10.5061/dryad.r4f8v).

## Electronic supplementary material

Supporting Information

## References

[1] Jansson R, Dynesius M (2002). The fate of clades in a world of recurrent climatic change: Milankovitch oscillations and evolution. Annual Review of Ecology and Systematics.

[2] Briggs JC (1987). Antitropical distribution and evolution in the Indo-West Pacific Ocean. Systematic Biology.

[3] Briggs JC (1987). Antitropicality and vicariance. Systematic Biology.

[4] Henriques R, Potts WM, Santos CV, Sauer WHH, Shaw PW (2014). Population connectivity and phylogeography of a coastal fish, *Atractoscion aequidens* (Sciaenidae), across the Benguela current region: evidence of an ancient vicariant event. Plos One.

[5] Rocha LA, Bowen BW (2008). Speciation in coral-reef fishes. Journal of Fish Biology.

[6] Palumbi SR (1992). Marine speciation on a small planet. Trends in Ecology and Evolution.

[7] Mirams AGK, Treml EA, Shields JL, Liggins L, Riginos C (2011). Vicariance and dispersal across an intermittent barrier: population genetic structure of marine animals across the Torres Strait land bridge. Coral Reefs.

[8] Burridge CP (2002). Antitropicality of Pacific fishes: molecular insights. Environmental Biology of Fishes.

[9] Bowen BW, Muss A, Rocha LA, Grant WS (2006). Shallow mtDNA coalescence in Atlantic pygmy angelfishes (genus *Centropyge*) indicates a recent invasion from the Indian Ocean. Journal of Heredity.

[10] Whitehead, P. J., Nelson, W. S. & Wongratana, T. *Clupeoid fishes of the World (Suborder Clupeoidei)*. Vol. 7 (FAO, 1988).

[11] Borsa P, Collet A, Durand JD (2004). Nuclear-DNA markers confirm the presence of two anchovy species in the Mediterranean. Comptes Rendus Biologies.

[12] Montes I (2016). Transcriptome analysis deciphers evolutionary mechanisms underlying genetic differentiation between coastal and offshore anchovy populations in the Bay of Biscay. Marine Biology.

[13] Grant WS, Lecomte F, Bowen BW (2010). Biogeographical contingency and the evolution of tropical anchovies (genus *Cetengraulis*) from temperate anchovies (genus *Engraulis*). Journal of Biogeography.

[14] Silva G, Horne JB, Castilho R (2014). Anchovies go north and west without losing diversity: post-glacial range expansions in a small pelagic fish. Journal of Biogeography.

[15] Grant WS, Leslie RW, Bowen BW (2005). Molecular genetic assessment of bipolarity in the anchovy genus *Engraulis*. Journal of Fish Biology.

[16] Grant WS, Bowen BW (2006). Living in a tilted world: climate change and geography limit speciation in Old World anchovies (*Engraulis*; Engraulidae). Biological Journal of the Linnean Society.

[17] Le Moan A, Gagnaire PA, Bonhomme F (2016). Parallel genetic divergence among coastal–marine ecotype pairs of European anchovy explained by differential introgression after secondary contact. Molecular Ecology.

[18] Jemaa S, Bacha M, Khalaf G, Amara R (2015). Evidence for population complexity of the European anchovy (*Engraulis encrasicolus*) along its distributional range. Fisheries Research.

[19] Kristoffersen JB, Magoulas A (2008). Population structure of anchovy *Engraulis encrasicolus* L. in the Mediterranean Sea inferred from multiple methods. Fisheries Research.

[20] Magoulas A, Castilho R, Caetano S, Marcato S, Patarnello T (2006). Mitochondrial DNA reveals a mosaic pattern of phylogeographical structure in Atlantic and Mediterranean populations of anchovy (*Engraulis encrasicolus*). Molecular Phylogenetics and Evolution.

[21] Sanz N, Garcia-Marín JL, Viñas J, Roldán M, Pla C (2008). Spawning groups of European anchovy: population structure and management implications. ICES Journal of Marine Science.

[22] Viñas, J. *et al*. Genetic population structure of European anchovy in the Mediterranean Sea and the northeast Atlantic Ocean using sequence analysis of the mitochondrial DNA control region. *ICES Journal of Marine Science* **7**, 391–397, doi:10.1093/icesjms/fst132 (2013).

[23] Silva, G., Lima, F. P., Martel, P. & Castilho, R. Thermal adaptation and clinal mitochondrial DNA variation of European anchovy. *Proceedings of the Royal Society of London B: Biological Sciences* **281**, 20141093, doi:10.1098/rspb.2014.1093 (2014). 10.1098/rspb.2014.1093 PMC4150322 25143035

[24] Liu JX (2006). Late Pleistocene divergence and subsequent population expansion of two closely related fish species, Japanese anchovy (*Engraulis japonicus*) and Australian anchovy (*Engraulis australis*). Molecular Phylogenetics and Evolution.

[25] Duchene D, Klanten SO, Munday PL, Herler J, van Herwerden L (2013). Phylogenetic evidence for recent diversification of obligate coral-dwelling gobies compared with their host corals. Molecular Phylogenetics and Evolution.

[26] Cárdenas L (2005). Origin, diversification, and historical biogeography of the genus *Trachurus* (Perciformes: Carangidae). Molecular Phylogenetics and Evolution.

[27] DiBattista J (2012). Twisted sister species of pygmy angelfishes: discordance between taxonomy, coloration, and phylogenetics. Coral Reefs.

[28] Horne JB, van Herwerden L, Choat JH, Robertson DR (2008). High population connectivity across the Indo-Pacific: congruent lack of phylogeographic structure in three reef fish congeners. Molecular Phylogenetics and Evolution.

[29] Reece JS, Bowen BW, Smith DG, Larson A (2011). Comparative phylogeography of four Indo-Pacific moray eel species (Muraenidae) reveals comparable ocean-wide genetic connectivity despite five-fold differences in available adult habitat. Marine Ecology Progress Series.

[30] Schwartzlose RA (1999). Worldwide large-scale fluctuations of sardine and anchovy populations. South African Journal of Marine Science.

[31] Grant WS, Bowen BW (1998). Shallow population histories in deep evolutionary lineages of marine fishes: insights from sardines and anchovies and lessons for conservation. The Journal of Heredity.

[32] Lessios HA, Robertson DR (2013). Speciation on a round planet: phylogeography of the goatfish genus Mulloidichthys. Journal of Biogeography.

[33] Sigman DM, Hain MP (2012). The biological productivity of the ocean. Nature Education Knowledge.

[34] Marlow JR, Lange CB, Wefer G, Rosell-Melé A (2000). Upwelling intensification as part of the Pliocene-Pleistocene climate transition. Science.

[35] Bloom, D. D. & Lovejoy, N. R. The evolutionary origins of diadromy inferred from a time-calibrated phylogeny for Clupeiformes (herring and allies). *Proceedings of the Royal Society B: Biological Sciences* **281**, doi:10.1098/rspb.2013.2081 (2014). 10.1098/rspb.2013.2081 PMC3906930 24430843

[36] Jombart T (2008). Adegenet: a R package for the multivariate analysis of genetic markers. Bioinformatics.

[37] R Development Core Team. R: A language and environment for statistical computing. (2013).

[38] Zarraonaindia I, Pardo MA, Iriondo M, Manzano C, Estonba A (2009). Microsatellite variability in European anchovy (*Engraulis encrasicolus*) calls for further investigation of its genetic structure and biogeography. ICES Journal of Marine Science.

[39] Ryman N, Palm S (2006). POWSIM: a computer program for assessing statistical power when testing for genetic differentiation. Molecular Ecology Notes.

[40] Geneious v. 5.1. (Biomatters Ltd, available at: http://www.geneious.com, Auckland, New Zealand), (2011).

[41] Posada, D. *Collapse 1.2: Describing haplotypes from sequence alignments* http://darwin.uvigo.es/software/collapse.html (2004).

[42] Excoffier L, Lischer HEL (2010). Arlequin suite ver 3.5: a new series of programs to perform population genetics analyses under Linux and Windows. Molecular Ecology Resources.

[43] Meirmans PG, Van Tienderen PH (2004). GENOTYPE and GENODIVE: two programs for the analysis of genetic diversity of asexual organisms. Molecular Ecology Notes.

[44] Tamura K (2011). MEGA5: Molecular Evolutionary Genetics Analysis using maximum likelihood, evolutionary distance, and maximum parsimony methods. Molecular Biology and Evolution.

[45] Teacher AGF, Griffiths DJ (2011). HapStar: automated haplotype network layout and visualization. Molecular Ecology Resources.

[46] Hedrick PW, Goodnight C (2005). A standardized genetic differentiation measure. Evolution.

[47] Jost L (2008). G(ST) and its relatives do not measure differentiation. Molecular Ecology.

[48] Pennings PS, Achenbach A, Foitzik S (2011). Similar evolutionary potentials in an obligate ant parasite and its two host species. Journal of Evolutionary Biology.

[49] Keenan K, McGinnity P, Cross TF, Crozier WW, Prodöhl P (2013). A. diveRsity: An R package for the estimation and exploration of population genetics parameters and their associated errors. Methods in Ecology and Evolution.

[50] Beerli P, Felsenstein J (1999). Maximum likelihood estimation of migration rates and effective population numbers in two populations using a coalescent approach. Genetics.

[51] Beerli P, Felsenstein J (2001). Maximum likelihood estimation of a migration matrix and effective population sizes in n subpopulations by using a coalescent approach. Proceedings of the National Academy of Sciences.

[52] Ivanova, E. V. The global thermohaline paleocirculation. (Springer, 2009).

[53] Lutjeharms, J. R. E. In *The sea: the global coastal ocean* Vol. 14B *The sea - ideas and observations on the progress in the study of the seas* (eds Allan, R. Robinson & Kenneth, Brink) 783–834 (Harvard University Press, 2006).

[54] Beerli P, Palczewski M (2010). Unified framework to evaluate panmixia and migration direction among multiple sampling locations. Genetics.

[55] Bloomquist, E. W., Lemey, P. & Suchard, M. A. Three roads diverged? Routes to phylogeographic inference. *Trends in Ecology & Evolution* **25**, 626–632, 10.1016/j.tree.2010.08.010 (2010). 10.1016/j.tree.2010.08.010 PMC2956787 20863591

[56] Lavoué S, Miya M, Nishida M (2010). Mitochondrial phylogenomics of anchovies (family Engraulidae) and recurrent origins of pronounced miniaturization in the order Clupeiformes. Molecular Phylogenetics and Evolution.

[57] Akaike H (1974). A new look at the statistical model identifications. IEEE Transactions on automatic control.

[58] Posada D, Crandall KA (1998). Modeltest: testing the model of DNA substituition. Bioinformatics.

[59] Ronquist, F. *et al*. MrBayes 3.2: efficient Bayesian phylogenetic inference and model choice across a large model space. *Systematic Biology* **61**, 539–542, doi:10.1093/sysbio/sys029 (2012). 10.1093/sysbio/sys029 PMC3329765 22357727

[60] Guindon S (2010). New Algorithms and Methods to Estimate Maximum-Likelihood Phylogenies: Assessing the Performance of PhyML 3.0. Systematic Biology.

[61] Bouckaert R (2014). BEAST 2: a software platform for Bayesian evolutionary analysis. PLoS Comput Biol.

[62] Gernhard T (2008). The conditioned reconstructed process. J Theor Biol.

[63] Grande, L. & Nelson, G. Interrelationships of fossil and recent anchovies (Teleostei: Engrauloidea) and description of a new species from the Miocene of Cyprus. *American Museum Novitates* **2826**, 1–16 (1985).

[64] Lessios HA, Kessing BD, Robertson DR, Paulay G (1999). Phylogeography of the pantropical sea urchin *Eucidaris* in relation to land barriers and ocean currents. Evolution.

[65] Yabumoto, Y. Pleistocene clupeid and engraulidid fishes from Kokubu group in Kagoshima Prefecture, Japan. *Bulletin of Kitakyushu Museum Natural History* **8**, 55–74 (1988).

[66] Rambaut, A., Suchard, M. A., Xie, D. & Drummond, A. J. Tracer v1.6. *Available from* http://beast.bio.ed.ac.uk/Tracer (2014).

