# Supplementary material for: Wandering behaviour prevents inter and intra oceanic speciation in a coastal pelagic fish
Source: Sci Rep. 2017 Jun 6;7:2893. doi: 10.1038/s41598-017-02945-0 (PMC5460251; doi:10.1038/s41598-017-02945-0)
Supplement: Supplementary file 1 — Supporting Information [file 41598_2017_2945_MOESM1_ESM.pdf]

## Supporting Information

### Wandering behaviour prevents inter and intra oceanic speciation in a coastal pelagic fish

Gonçalo Silva, Regina L. Cunha, Ana Ramos and Rita Castilho

**Table S1 -** Summary statistics across eight microsatellite loci of the Old World Anchovies. $A_{rang}$, allele range (bp); $\boldsymbol{A_r}$, number of alleles across locations; *Effnum*, effective number of alleles; $H_O$, observed heterozygosity; $H_E$, expected heterozygosity; *H*t, total heterozygosity; *H*'t, corrected total heterozygosity; $G_{IS}$, inbreeding coefficient.

| **Nuclear microsatellites** | **Genetic diversity** | | | | | | | | |
|---|---|---|---|---|---|---|---|---|---|
| **Name (acc. no.)** | $A_{rang}$ | $A_r$ | $A_r$=9 | ***Effnum*** | $H_O$ | $H_E$ | ***H*t** | ***H*'t** | $G_{IS}$ |
| Ee10 (AY241273) | 197-381 | 85 | 62 | 8.179 | 0.854 | 0.895 | 0.947 | 0.950 | 0.045 |
| Ee2-135 (FJ534738) | 102-170 | 24 | 17 | 5.906 | 0.758 | 0.848 | 0.870 | 0.871 | 0.107 |
| Ee2-407 (FJ534751) | 128-296 | 62 | 38 | 7.292 | 0.816 | 0.880 | 0.922 | 0.925 | 0.073 |
| Ee2-452a (FJ534754) | 245-357 | 55 | 37 | 11.139 | 0.886 | 0.929 | 0.951 | 0.952 | 0.047 |
| Ee2-508 (FJ534759) | 154-198 | 29 | 26 | 7.213 | 0.589 | 0.884 | 0.927 | 0.930 | 0.334 |
| Ee2-91a (FJ534732) | 197-298 | 39 | 19 | 6.451 | 0.613 | 0.866 | 0.903 | 0.906 | 0.292 |
| Ee2-91b (FJ534732) | 100-165 | 19 | 13 | 4.851 | 0.809 | 0.809 | 0.818 | 0.818 | -0.000 |
| EJ41.1 (AF344659) | 140-283 | 68 | 44 | 4.941 | 0.644 | 0.816 | 0.866 | 0.869 | 0.211 |

Primers information and PCR conditions in Silva *et al.* [1]

**Table S2** – GenBank accession numbers of cytochrome *b* fragments of specimens used in Bayesian and Maximum Likelihood phylogenetic analyses.

| Species | Accession Number | Family | Sub-family | Native |
|---|---|---|---|---|
| *Coilia brachygnathus* | EU694410.1 | Engraulidae | Coilinae | Pacific |
| *Coilia ectenes* | NC_019625.1 | Engraulidae | Coilinae | Pacific |
| *Coilia lindmani* | AP011558.1 | Engraulidae | Coilinae | Indo-Pacific |
| *Coilia mystus* | EU694407.1 | Engraulidae | Coilinae | Pacific |
| *Coilia nasus* | EU694403.1 | Engraulidae | Coilinae | Pacific |
| *Coilia reynaldi* | AP011559.1 | Engraulidae | Coilinae | Indo-Pacific |
| *Lycothrissa crocodilus* | JQ012420.1 | Engraulidae | Coilinae | Indo-Pacific |
| *Setipinna melanochir* | AP011565.1 | Engraulidae | Coilinae | Indo-Pacific |
| *Setipinna taty* | JQ012365.1 | Engraulidae | Coilinae | Indian |
| *Setipinna tenuifilis* | JQ012398.1 | Engraulidae | Coilinae | Indo-Pacific |
| *Thryssa baelama* | AP009616.1 | Engraulidae | Coilinae | Indo-Pacific |
| *Thryssa dussumieri* | JQ012363.1 | Engraulidae | Coilinae | Indo-Pacific |
| *Thryssa mystax* | JQ012366.1 | Engraulidae | Coilinae | Indo-Pacific |
| *Amazonsprattus scintilla* | JQ012351.1 | Engraulidae | Engraulinae | Atlantic |
| *Anchoa cayorum* | JQ012346.1 | Engraulidae | Engraulinae | Atlantic |
| *Anchoa chamensis* | JQ012375.1 | Engraulidae | Engraulinae | Pacific |
| *Anchoa colonensis* | JQ012383.1 | Engraulidae | Engraulinae | Atlantic |
| *Anchoa cubana* | JQ012342.1 | Engraulidae | Engraulinae | Atlantic |
| *Anchoa delicatissima* | JQ012348.1 | Engraulidae | Engraulinae | Pacific |
| *Anchoa filifera* | JQ012387.1 | Engraulidae | Engraulinae | Atlantic |
| *Anchoa lamprotaenia* | JQ012379.1 | Engraulidae | Engraulinae | Atlantic |
| *Anchoa lyolepis* | JQ012344.1 | Engraulidae | Engraulinae | Atlantic |
| *Anchoa mitchilli* | JQ012357.1 | Engraulidae | Engraulinae | Atlantic |
| *Anchoa mundeoloides* | JQ012419.1 | Engraulidae | Engraulinae | Pacific |
| *Anchoa nasus* | JQ012373.1 | Engraulidae | Engraulinae | Pacific |
| *Anchoa panamensis* | JQ012392.1 | Engraulidae | Engraulinae | Pacific |
| *Anchoa parva* | JQ012377.1 | Engraulidae | Engraulinae | Atlantic |
| *Anchoa schofieldi* | JQ012349.1 | Engraulidae | Engraulinae | Pacific |
| *Anchoa walkeri* | JQ012369.1 | Engraulidae | Engraulinae | Pacific |
| *Anchovia clupeoides* | EU552570.1 | Engraulidae | Engraulinae | Atlantic |
| *Anchovia macrolepidota* | JQ012394.1 | Engraulidae | Engraulinae | Pacific |
| *Anchovia surinamensis* | JQ012402.1 | Engraulidae | Engraulinae | Atlantic |
| *Anchoviella alleni* | JQ012333.1 | Engraulidae | Engraulinae | Atlantic |
| *Anchoviella balboae* | JQ012371.1 | Engraulidae | Engraulinae | Pacific |
| *Anchoviella brevirostris* | JQ012412.1 | Engraulidae | Engraulinae | Atlantic |
| *Anchoviella carrikeri* | JQ012330.1 | Engraulidae | Engraulinae | Atlantic |
| *Anchoviella elongata* | JQ012381.1 | Engraulidae | Engraulinae | Atlantic |
| *Anchoviella guianensis* | JQ012327.1 | Engraulidae | Engraulinae | Atlantic |
| *Anchoviella lepidentostole* | JQ012414.1 | Engraulidae | Engraulinae | Atlantic |
| *Cetengraulis edentulus* | JQ012385.1 | Engraulidae | Engraulinae | Atlantic |
| *Cetengraulis mysticetus* | JQ012390.1 | Engraulidae | Engraulinae | Pacific |
| *Encrasicholina devisi* | JQ012364.1 | Engraulidae | Engraulinae | Indian |
| *Engraulis anchoita* | JQ012416.1 | Engraulidae | Engraulinae | Atlantic |
| *Engraulis australis* | KJ007734 | Engraulidae | Engraulinae | Pacific |
| *Engraulis capensis* | KF601464 | Engraulidae | Engraulinae | Atlantic |
| *Engraulis encrasicolus* | JQ716644 | Engraulidae | Engraulinae | Atlantic |
| *Engraulis eurystole* | JQ716748 | Engraulidae | Engraulinae | Atlantic |
| *Engraulis japonicus* | KJ007642 | Engraulidae | Engraulinae | Pacific |
| *Engraulis mordax* | JQ012350.1 | Engraulidae | Engraulinae | Pacific |
| *Engraulis ringens* | JQ012426.1 | Engraulidae | Engraulinae | Pacific |
| *Jurengraulis juruensis* | JQ012329.1 | Engraulidae | Engraulinae | Atlantic |
| *Lycengraulis batesii* | JQ012326.1 | Engraulidae | Engraulinae | Atlantic |
| *Lycengraulis grossidens* | JQ012396.1 | Engraulidae | Engraulinae | Atlantic |
| *Lycengraulis poeyi* | JQ012370.1 | Engraulidae | Engraulinae | Pacific |
| *Pterengraulis atherinoides* | JQ012323.1 | Engraulidae | Engraulinae | Atlantic |
| *Stolephorus chinensis* | AP011566.1 | Engraulidae | Engraulinae | Indo-Pacific |
| *Stolephorus waitei* | AP011567.1 | Engraulidae | Engraulinae | Indo-Pacific |
| *Chirocentrus dorab* | AP006229.1 | Chirocentridae | | |
| *Clupea harengus* | NC_009577.1 | Clupeidae | | |
| *Sardina pilchardus* | NC_009592.1 | Clupeidae | | |
| *Denticeps clupeoides* | NC_007889.1 | Denticipitidae | | |
| *Ilisha africana* | NC_009584.1 | Pristigasteridae | | |
| *Sundasalanx mekongensis* | AP006232.1 | Sundasalangidae | | |

**Table S3** – GenBank accession numbers of both mitochondrial (cytochrome *b* and 16S) and nuclear (RAG1 and RAG2 introns) fragments of specimens used in the Bayesian dating analyses.

| | **Mitochondrial Region** | | **Nuclear intron** | | | | |
|---|---|---|---|---|---|---|---|
| **Species** | **Cyt *b*** | **16S** | **RAG1** | **RAG2** | **Family** | **Sub-family** | **Native** |
| *Amazonsprattus scintilla* | JQ012351.1 | JQ012456.1 | JQ012538.1 | JQ012667.1 | Engraulidae | Engraulinae | Atlantic |
| *Anchoa cayorum* | JQ012346.1 | JQ012451.1 | JQ012555.1 | JQ012700.1 | Engraulidae | Engraulinae | Atlantic |
| *Anchoa chamensis* | JQ012375.1 | JQ012480.1 | JQ012563.1 | JQ012718.1 | Engraulidae | Engraulinae | Pacific |
| *Anchoa colonensis* | JQ012383.1 | JQ012488.1 | JQ012559.1 | JQ012716.1 | Engraulidae | Engraulinae | Atlantic |
| *Anchoa cubana* | JQ012342.1 | JQ012447.1 | JQ012550.1 | JQ012705.1 | Engraulidae | Engraulinae | Atlantic |
| *Anchoa delicatissima* | JQ012348.1 | JQ012453.1 | JQ012557.1 | JQ012704.1 | Engraulidae | Engraulinae | Pacific |
| *Anchoa filifera* | JQ012387.1 | JQ012492.1 | JQ012542.1 | JQ012722.1 | Engraulidae | Engraulinae | Atlantic |
| *Anchoa lamprotaenia* | JQ012379.1 | JQ012484.1 | JQ012630.1 | JQ012696.1 | Engraulidae | Engraulinae | Atlantic |
| *Anchoa lyolepis* | JQ012344.1 | JQ012449.1 | JQ012573.1 | JQ012688.1 | Engraulidae | Engraulinae | Atlantic |
| *Anchoa mitchilli* | JQ012357.1 | JQ012462.1 | JQ012552.1 | JQ012698.1 | Engraulidae | Engraulinae | Atlantic |
| *Anchoa mundeoloides* | JQ012419.1 | JQ012524.1 | JQ012565.1 | JQ012715.1 | Engraulidae | Engraulinae | Pacific |
| *Anchoa nasus* | JQ012373.1 | JQ012478.1 | JQ012575.1 | JQ012690.1 | Engraulidae | Engraulinae | Pacific |
| *Anchoa panamensis* | JQ012392.1 | JQ012497.1 | JQ012570.1 | JQ012712.1 | Engraulidae | Engraulinae | Pacific |
| *Anchoa parva* | JQ012377.1 | JQ012482.1 | JQ012558.1 | JQ012702.1 | Engraulidae | Engraulinae | Atlantic |
| *Anchoa schofieldi* | JQ012349.1 | JQ012454.1 | JQ012571.1 | JQ012711.1 | Engraulidae | Engraulinae | Pacific |
| *Anchoa spinifer* | KJ158085.1 | KJ158123.1 | KJ158140.1 | KJ158104.1 | Engraulidae | Engraulinae | Atlantic/ Pacific |
| *Anchoa walkeri* | JQ012369.1 | JQ012474.1 | JQ012568.1 | JQ012713.1 | Engraulidae | Engraulinae | Pacific |
| *Anchovia clupeoides* | KJ158087.1 | KJ158125.1 | KJ158142.1 | KJ158106.1 | Engraulidae | Engraulinae | Atlantic |
| *Anchovia macrolepidota* | JQ012394.1 | JQ012499.1 | JQ012561.1 | JQ012709.1 | Engraulidae | Engraulinae | Pacific |
| *Anchovia surinamensis* | JQ012402.1 | JQ012507.1 | JQ012613.1 | JQ012665.1 | Engraulidae | Engraulinae | Atlantic |
| *Anchoviella alleni* | JQ012334.1 | JQ012439.1 | JQ012607.1 | JQ012656.1 | Engraulidae | Engraulinae | Atlantic |
| *Anchoviella balboae* | JQ012371.1 | JQ012476.1 | JQ012566.1 | JQ012720.1 | Engraulidae | Engraulinae | Pacific |
| *Anchoviella brevirostris* | JQ012412.1 | JQ012517.1 | JQ012608.1 | JQ012686.1 | Engraulidae | Engraulinae | Atlantic |
| *Anchoviella carrikeri* | JQ012330.1 | JQ012435.1 | JQ012604.1 | JQ012658.1 | Engraulidae | Engraulinae | Atlantic |
| *Anchoviella guianensis* | JQ012400.1 | JQ012505.1 | JQ012585.1 | JQ012673.1 | Engraulidae | Engraulinae | Atlantic |
| *Anchoviella elongata* | JQ012381.1 | JQ012486.1 | JQ012548.1 | JQ012707.1 | Engraulidae | Engraulinae | Atlantic |
| *Anchoviella lepidentostole* | JQ012414.1 | JQ012519.1 | JQ012596.1 | JQ012634.1 | Engraulidae | Engraulinae | Atlantic |

| | | | | | | | |
|---|---|---|---|---|---|---|---|
| *Cetengraulis edentulus* | JQ012385.1 | JQ012490.1 | JQ012577.1 | JQ012692.1 | Engraulidae | Engraulinae | Atlantic |
| *Cetengraulis mysticetus* | JQ012390.1 | JQ012495.1 | JQ012579.1 | JQ012694.1 | Engraulidae | Engraulinae | Pacific |
| *Coilia brachygnathus* | EU694410.1 | DQ912089.1 | DQ912124.1 | DQ912159.1 | Engraulidae | Coilinae | Pacific |
| *Coilia mystus* | JX030395.1 | DQ912092.1 | DQ912126.1 | DQ912162.1 | Engraulidae | Coilinae | Pacific |
| *Coilia nasus* | EU694403.1 | DQ912087.1 | DQ912123.1 | DQ912157.1 | Engraulidae | Coilinae | Pacific |
| *Encrasicholina devisi* | JQ012364.1 | JQ012469.1 | JQ012626.1 | JQ012684.1 | Engraulidae | Engraulinae | Indian |
| *Engraulis australis* | This study | This study | This study | This study | Engraulidae | Engraulinae | Pacific |
| *Engraulis encrasicolus B 1844* | This study | This study | This study | This study | Engraulidae | Engraulinae | Atlantic |
| *Engraulis encrasicolus B 1847* | This study | This study | This study | This study | Engraulidae | Engraulinae | Atlantic |
| *Engraulis encrasicolus A* | JQ012359.1 | DQ912066.1 | JQ012540.1 | JQ012726.1 | Engraulidae | Engraulinae | Atlantic |
| *Engraulis japonicus* | AB374208.1 | KF765500.2 | AY430205.1 | This study | Engraulidae | Engraulinae | Pacific |
| *Engraulis mordax* | JQ012350.1 | JQ012455.1 | JQ012546.1 | JQ012728.1 | Engraulidae | Engraulinae | Pacific |
| *Engraulis ringens* | JQ012426.1 | JQ012532.1 | JQ012595.1 | JQ012731.1 | Engraulidae | Engraulinae | Pacific |
| *Jurengraulis juruensis* | JQ012329.1 | JQ012434.1 | JQ012610.1 | JQ012732.1 | Engraulidae | Engraulinae | Atlantic |
| *Lycengraulis batesii* | JQ012410.1 | JQ012515.1 | JQ012619.1 | JQ012643.1 | Engraulidae | Engraulinae | Atlantic |
| *Lycengraulis grossidens* | JQ012396.1 | JQ012501.1 | JQ012622.1 | JQ012639.1 | Engraulidae | Engraulinae | Atlantic |
| *Lycengraulis poeyi* | JQ012370.1 | JQ012475.1 | JQ012621.1 | JQ012642.1 | Engraulidae | Engraulinae | Pacific |
| *Pterengraulis atherinoides* | JQ012323.1 | JQ012428.1 | JQ012616.1 | JQ012636.1 | Engraulidae | Engraulinae | Atlantic |
| *Setipinna taty* | JQ012365.1 | DQ912091.1 | DQ912125.1 | DQ912161.1 | Engraulidae | Coilinae | Pacific |
| *Stolephorus sp.* | JQ012361.1 | JQ012466.1 | JQ012536.1 | JQ012671.1 | Engraulidae | Engraulinae | Indo-Pacific |
| *Thryssa dussumieri* | JQ012368.1 | JQ012473.1 | JQ012625.1 | JQ012679.1 | Engraulidae | Coilinae | Indo-Pacific |
| *Thryssa mystax* | JQ012366.1 | JQ012471.1 | JQ012537.1 | JQ012680.1 | Engraulidae | Coilinae | Indo-Pacific |
| *Chirocentrus dorab* | AP006229.1\| | AP006229.1 | DQ912127.1 | DQ912163.1 | Chirocentridae | - | - |
| *Clupea harengus* | AB278564.1\| | DQ912078.1\| | DQ912114.1 | DQ912148.1 | Clupeidae | - | - |
| *Denticeps clupeoides* | EU552629.1\| | DQ912063.1\| | DQ912100.1 | DQ912133.1 | Denticipitidae | - | - |

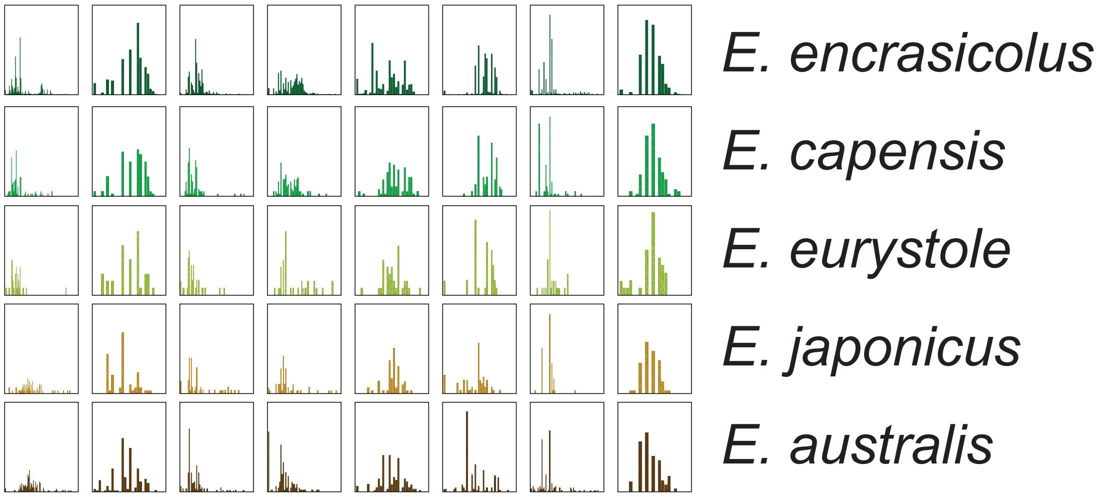


**Figure S1** - Distribution of nuclear microsatellite alleles per locus (columns) and per putative species (rows).

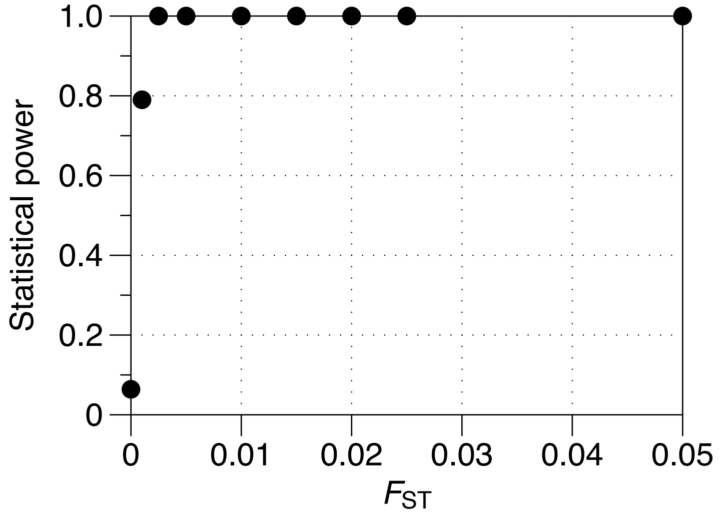


**Figure S2** - POWSIM analysis showing power to detect pairwise genetic differentiation of subpopulations of Old World Anchovies corresponding to (1) European anchovy (*Engraulis encrasicolus*, *E. capensis* and *E. eurystole*), (2) *E. japonicus* and (3) *E. australis*, from a single ancestral population at different $F_{ST}$ levels.

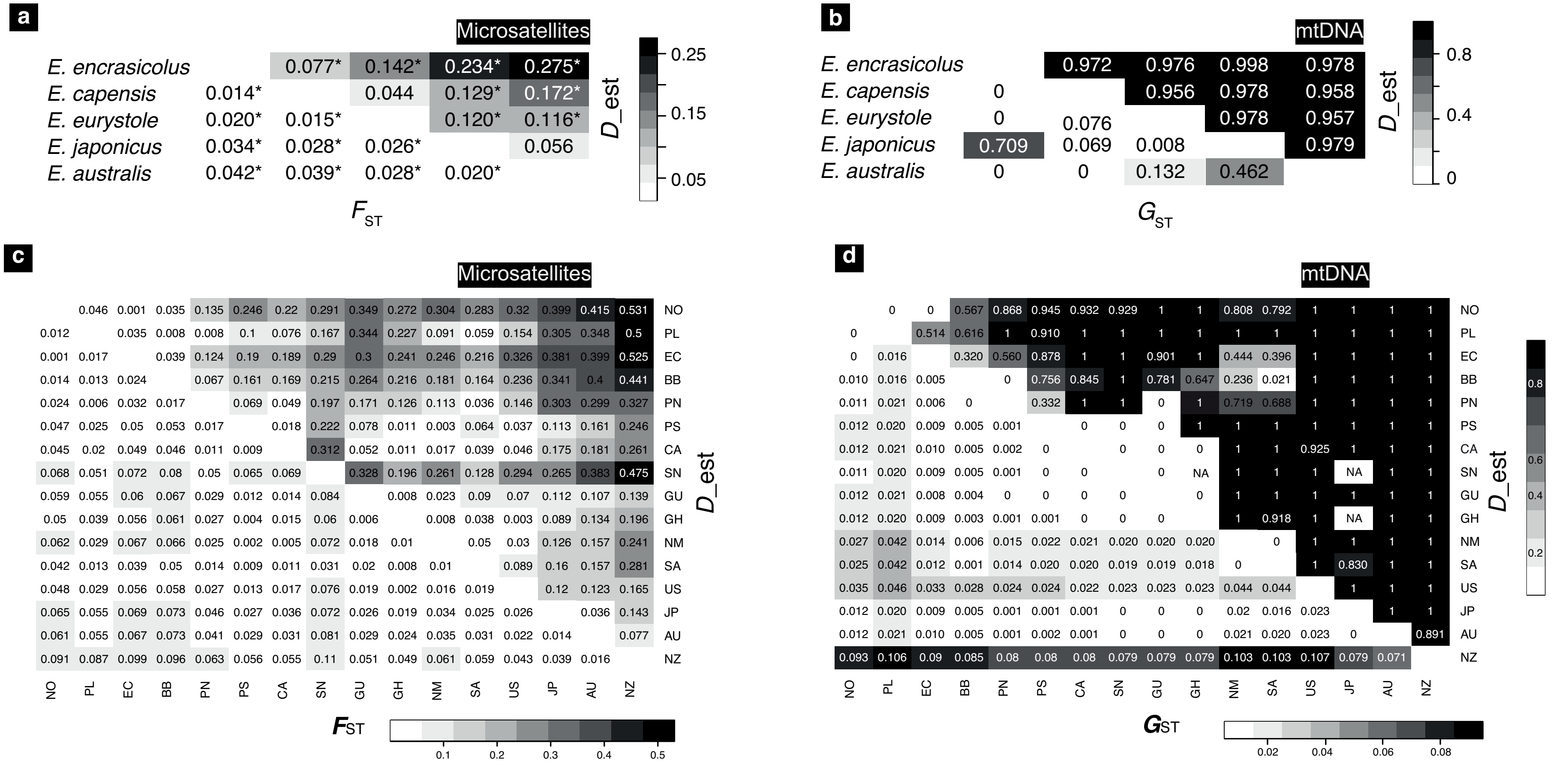


**Figure S3** - a) Pairwise-taxa $F_{st}$ (below diagonal) and $D_{est}$ values (above diagonal) for microsatellites; b) Pairwise-taxa $G_{st}$ (below diagonal) and $D_{est}$ values (above diagonal) for mtDNA; c) Pairwise-locations $F_{st}$ (below diagonal) and $D_{est}$ values (above diagonal) for microsatellites; d) Pairwise-locations $G_{st}$ (below diagonal) and $D_{est}$ values (above diagonal) for mtDNA; site abbreviations defined in Table 1.

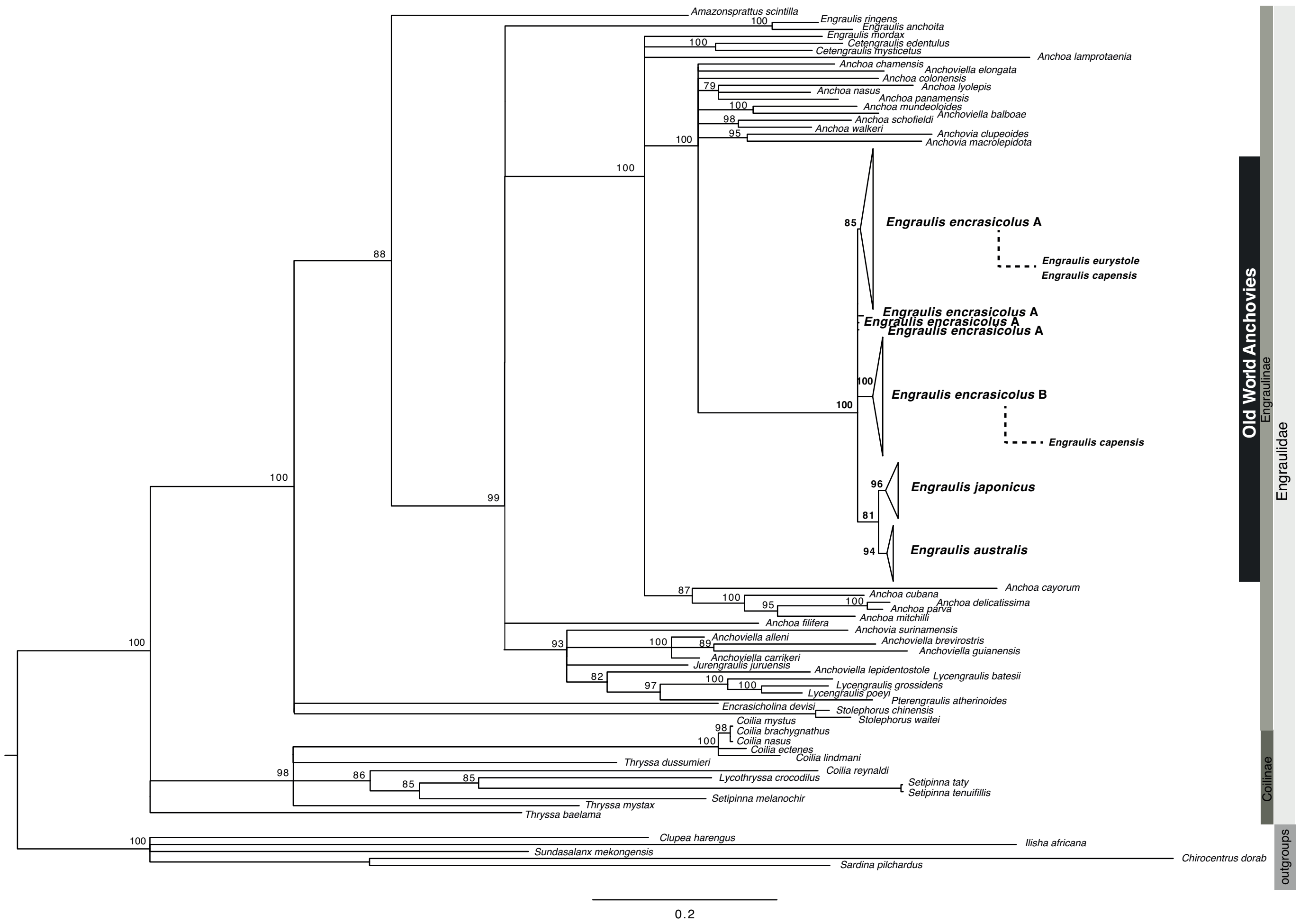


**Figure S4** - Phylogenetic relationships of Old World Anchovies *Engraulis spp*. based on Bayesian inference inferred from a fragment of 1044bp of cyt *b*. Bayesian posterior probabilities (BPP) for major supported clades larger than 75 are shown above branches.

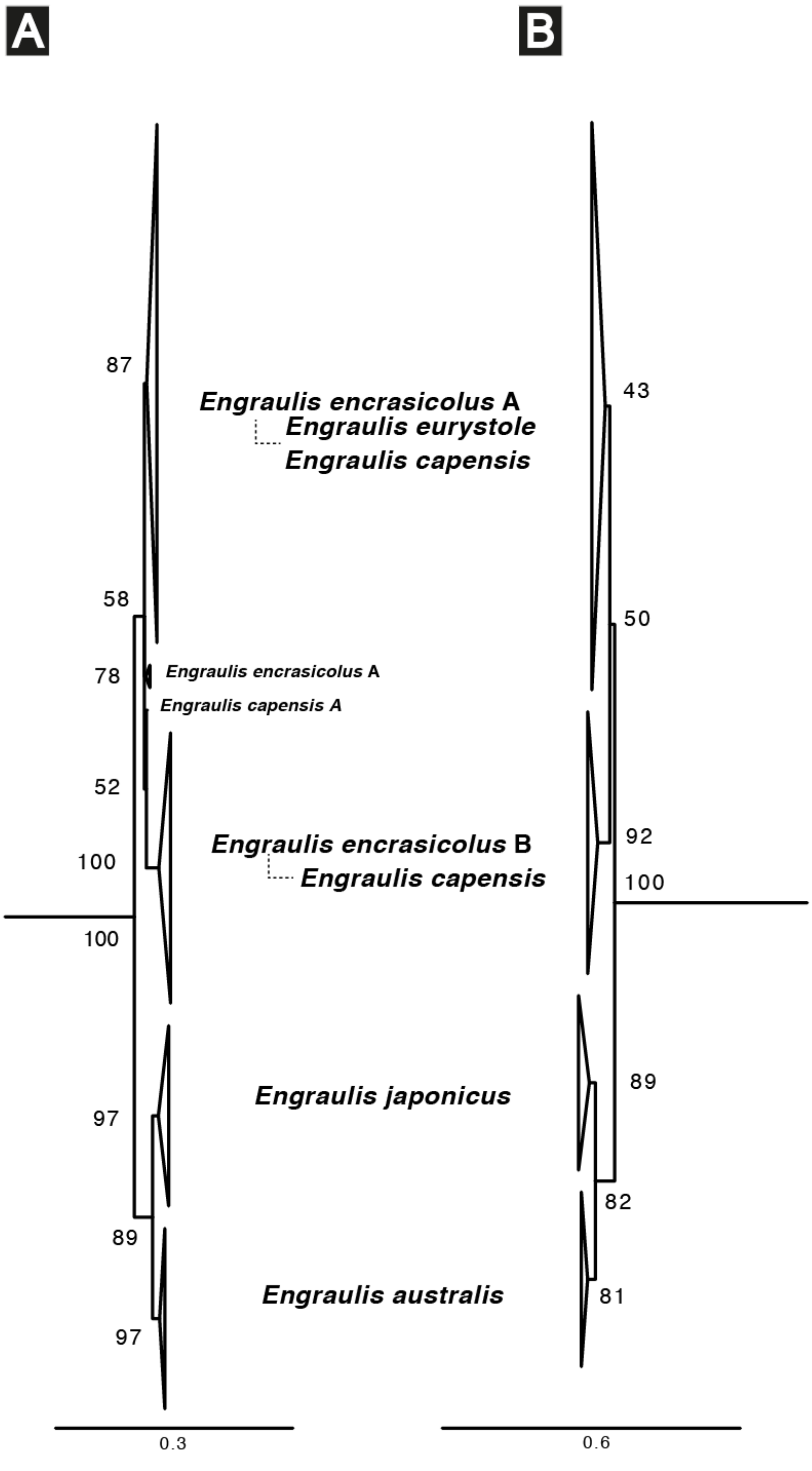


**Figure S5 -** Zoom of the phylogenetic relationships of Old World Anchovies *Engraulis spp*. based on Bayesian inference (A) and maximum likelihood analyses (B) inferred from a fragment of 1044bp of cyt *b*. All individuals of *E. encrasicolus*/ *E. capensis* clade B presented here are under selection in codon 368[2]. Bayesian posterior probabilities (BPP) and maximum-likelihood bootstrap values are shown above branches.

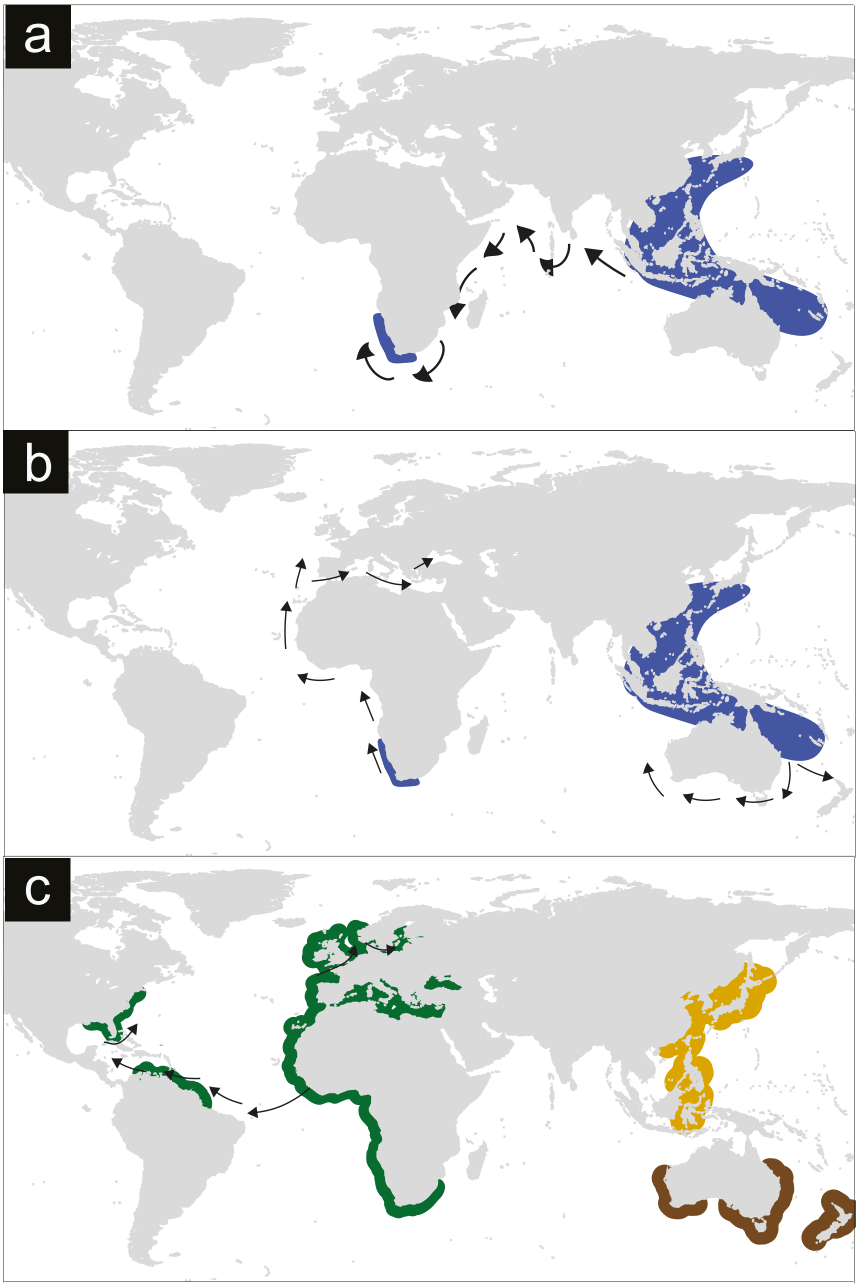


**Figure S6** - Hypothetical biogeographic scenario of Old World anchovies (OWA); a) colonisation route of the OWA from the Pacific to the Atlantic Ocean before 0.670 Myr; blue shadings represent putative distribution of ancestral populations; b) trans-Equatorial dispersals in each oceanic basin after 0.670 Myr; c) present-day distribution of OWA, with post-LGM trans-Atlantic dispersal and northeastern Atlantic colonisation after ice sheets retreat around 8 ka. Raw maps were downloaded from https://freevectormaps.com/ and edited in Adobe Illustrator CS5.1 (Adobe Systems Inc., CA, USA).

## References

1 Silva, G., Horne, J. B. & Castilho, R. Anchovies go north and west without losing diversity: post-glacial range expansions in a small pelagic fish. *Journal of Biogeography* **41**, 1171-1182, doi:10.1111/jbi.12275 (2014).

2 Silva, G., Lima, F. P., Martel, P. & Castilho, R. Thermal adaptation and clinal mitochondrial DNA variation of European anchovy. *Proceedings of the Royal Society of London B: Biological Sciences* **281** (2014).
